# Supplementary material for: Modeling glioblastoma heterogeneity as a dynamic network of cell states
Source: Mol Syst Biol. 2021 Sep 16;17(9):e10105. doi: 10.15252/msb.202010105 (PMC8444284; doi:10.15252/msb.202010105)
Supplement: Supplementary file 5 — Source Data for Figure 3 [file MSB-17-e10105-s001.zip › Figure3A_sourcedata/GSEA_3065/nosigdb_state6.GseaPreranked.1622718443993/LARSSON_STATE_6.html]

Details for gene set LARSSON\_STATE\_6[GSEA]

|  || Dataset | state6 |
| Phenotype | NoPhenotypeAvailable |
| Upregulated in class | na\_pos |
| GeneSet | LARSSON\_STATE\_6 |
| Enrichment Score (ES) | 0.6457666 |
| Normalized Enrichment Score (NES) | 2.7533777 |
| Nominal p-value | 0.0 |
| FDR q-value | 0.0 |
| FWER p-Value | 0.0 |
Table: GSEA Results Summary

  

Fig 1: Enrichment plot: LARSSON\_STATE\_6      
 Profile of the Running ES Score & Positions of GeneSet Members on the Rank Ordered List

  

| PROBE | GENE SYMBOL | GENE\_TITLE | RANK IN GENE LIST | RANK METRIC SCORE | RUNNING ES | CORE ENRICHMENT || 1 | SOX4 |  |  | 3 | 0.375 | 0.0278 | Yes |
| 2 | EEF1A1 |  |  | 9 | 0.325 | 0.0516 | Yes |
| 3 | RPS27 |  |  | 11 | 0.307 | 0.0745 | Yes |
| 4 | C9orf16 |  |  | 13 | 0.300 | 0.0968 | Yes |
| 5 | RAB31 |  |  | 23 | 0.284 | 0.1170 | Yes |
| 6 | ZMAT3 |  |  | 40 | 0.252 | 0.1340 | Yes |
| 7 | RPL34 |  |  | 41 | 0.251 | 0.1528 | Yes |
| 8 | TPT1 |  |  | 48 | 0.247 | 0.1706 | Yes |
| 9 | OST4 |  |  | 50 | 0.246 | 0.1889 | Yes |
| 10 | FTH1 |  |  | 52 | 0.245 | 0.2071 | Yes |
| 11 | FHL1 |  |  | 61 | 0.236 | 0.2238 | Yes |
| 12 | FTL |  |  | 64 | 0.234 | 0.2412 | Yes |
| 13 | RTL8C |  |  | 66 | 0.232 | 0.2585 | Yes |
| 14 | RPS11 |  |  | 85 | 0.221 | 0.2730 | Yes |
| 15 | PHPT1 |  |  | 89 | 0.218 | 0.2890 | Yes |
| 16 | SERF2 |  |  | 97 | 0.215 | 0.3043 | Yes |
| 17 | BRI3 |  |  | 122 | 0.202 | 0.3167 | Yes |
| 18 | PNRC1 |  |  | 123 | 0.202 | 0.3318 | Yes |
| 19 | CCNI |  |  | 127 | 0.201 | 0.3465 | Yes |
| 20 | SOX9 |  |  | 148 | 0.193 | 0.3587 | Yes |
| 21 | TTC3 |  |  | 160 | 0.188 | 0.3715 | Yes |
| 22 | CRYAB |  |  | 173 | 0.184 | 0.3839 | Yes |
| 23 | TXNIP |  |  | 176 | 0.183 | 0.3974 | Yes |
| 24 | SRP14 |  |  | 181 | 0.182 | 0.4106 | Yes |
| 25 | NLRP1 |  |  | 203 | 0.175 | 0.4213 | Yes |
| 26 | MARCKSL1 |  |  | 223 | 0.169 | 0.4317 | Yes |
| 27 | PFDN5 |  |  | 224 | 0.168 | 0.4443 | Yes |
| 28 | GFAP |  |  | 226 | 0.168 | 0.4568 | Yes |
| 29 | BNIP3L |  |  | 240 | 0.164 | 0.4676 | Yes |
| 30 | C1QL1 |  |  | 267 | 0.158 | 0.4764 | Yes |
| 31 | PTMS |  |  | 269 | 0.157 | 0.4881 | Yes |
| 32 | C4orf3 |  |  | 286 | 0.153 | 0.4977 | Yes |
| 33 | LAMTOR4 |  |  | 295 | 0.152 | 0.5082 | Yes |
| 34 | SNX3 |  |  | 306 | 0.150 | 0.5183 | Yes |
| 35 | TOMM7 |  |  | 310 | 0.149 | 0.5291 | Yes |
| 36 | COMMD6 |  |  | 350 | 0.144 | 0.5354 | Yes |
| 37 | S100B |  |  | 391 | 0.137 | 0.5411 | Yes |
| 38 | ATP6V1F |  |  | 407 | 0.135 | 0.5495 | Yes |
| 39 | C4orf48 |  |  | 441 | 0.129 | 0.5554 | Yes |
| 40 | MXD4 |  |  | 495 | 0.124 | 0.5586 | Yes |
| 41 | SESN3 |  |  | 533 | 0.120 | 0.5633 | Yes |
| 42 | RND2 |  |  | 534 | 0.120 | 0.5723 | Yes |
| 43 | ZNF428 |  |  | 553 | 0.118 | 0.5790 | Yes |
| 44 | CTXN1 |  |  | 589 | 0.115 | 0.5836 | Yes |
| 45 | MAP1LC3A |  |  | 607 | 0.113 | 0.5902 | Yes |
| 46 | BEX3 |  |  | 637 | 0.111 | 0.5952 | Yes |
| 47 | GABARAPL2 |  |  | 642 | 0.111 | 0.6030 | Yes |
| 48 | MT3 |  |  | 662 | 0.109 | 0.6090 | Yes |
| 49 | MARCH2 |  |  | 688 | 0.107 | 0.6141 | Yes |
| 50 | SH3BGRL |  |  | 701 | 0.105 | 0.6206 | Yes |
| 51 | SAT1 |  |  | 744 | 0.102 | 0.6235 | Yes |
| 52 | TUBB2B |  |  | 795 | 0.099 | 0.6251 | Yes |
| 53 | FYN |  |  | 817 | 0.097 | 0.6300 | Yes |
| 54 | QKI |  |  | 820 | 0.097 | 0.6370 | Yes |
| 55 | PFN2 |  |  | 942 | 0.089 | 0.6297 | Yes |
| 56 | TSC22D4 |  |  | 976 | 0.087 | 0.6324 | Yes |
| 57 | ID2 |  |  | 1058 | 0.082 | 0.6293 | Yes |
| 58 | SCP2 |  |  | 1067 | 0.082 | 0.6345 | Yes |
| 59 | MARCKS |  |  | 1081 | 0.081 | 0.6391 | Yes |
| 60 | CEBPD |  |  | 1166 | 0.077 | 0.6352 | Yes |
| 61 | PBX1 |  |  | 1168 | 0.077 | 0.6409 | Yes |
| 62 | CITED1 |  |  | 1214 | 0.075 | 0.6413 | Yes |
| 63 | ID3 |  |  | 1329 | 0.070 | 0.6335 | Yes |
| 64 | BEX1 |  |  | 1352 | 0.069 | 0.6361 | Yes |
| 65 | FKBP8 |  |  | 1394 | 0.068 | 0.6365 | Yes |
| 66 | METRN |  |  | 1396 | 0.068 | 0.6414 | Yes |
| 67 | SKP1 |  |  | 1403 | 0.067 | 0.6458 | Yes |
| 68 | PMP2 |  |  | 1515 | 0.063 | 0.6377 | No |
| 69 | C1orf122 |  |  | 1527 | 0.063 | 0.6412 | No |
| 70 | PLEKHB1 |  |  | 1590 | 0.061 | 0.6386 | No |
| 71 | TUBA1A |  |  | 2135 | 0.044 | 0.5793 | No |
| 72 | IRS2 |  |  | 2158 | 0.043 | 0.5800 | No |
| 73 | FOXO3 |  |  | 2178 | 0.042 | 0.5810 | No |
| 74 | ID1 |  |  | 2451 | 0.035 | 0.5523 | No |
| 75 | SEPT7 |  |  | 2478 | 0.034 | 0.5519 | No |
| 76 | NFIB |  |  | 2762 | 0.028 | 0.5214 | No |
| 77 | ATP6V1G1 |  |  | 2997 | 0.022 | 0.4961 | No |
| 78 | FEZ1 |  |  | 3090 | 0.020 | 0.4871 | No |
| 79 | HEY1 |  |  | 3541 | 0.011 | 0.4361 | No |
| 80 | SMDT1 |  |  | 3951 | 0.001 | 0.3892 | No |
| 81 | GNAI2 |  |  | 4005 | 0.001 | 0.3831 | No |
| 82 | MAGED1 |  |  | 4805 | -0.014 | 0.2923 | No |
| 83 | CTNNA1 |  |  | 5850 | -0.035 | 0.1749 | No |
| 84 | FABP7 |  |  | 6373 | -0.047 | 0.1184 | No |
| 85 | CD74 |  |  | 6826 | -0.059 | 0.0709 | No |
| 86 | HOPX |  |  | 6940 | -0.063 | 0.0626 | No |
| 87 | HNRNPDL |  |  | 6956 | -0.063 | 0.0656 | No |
| 88 | TUBB2A |  |  | 7078 | -0.067 | 0.0567 | No |
| 89 | TNFRSF21 |  |  | 7096 | -0.068 | 0.0598 | No |
| 90 | EFTUD2 |  |  | 7238 | -0.072 | 0.0490 | No |
| 91 | LGALS3 |  |  | 7566 | -0.084 | 0.0178 | No |
| 92 | DKK3 |  |  | 7776 | -0.094 | 0.0008 | No |
| 93 | OLFM2 |  |  | 7907 | -0.102 | -0.0066 | No |
| 94 | HNRNPA2B1 |  |  | 7941 | -0.103 | -0.0026 | No |
| 95 | SPARCL1 |  |  | 8104 | -0.114 | -0.0127 | No |
| 96 | CLU |  |  | 8387 | -0.145 | -0.0343 | No |
| 97 | FABP5 |  |  | 8414 | -0.148 | -0.0262 | No |
| 98 | FAM20C |  |  | 8424 | -0.149 | -0.0161 | No |
| 99 | COL4A3BP |  |  | 8516 | -0.165 | -0.0141 | No |
| 100 | GABBR2 |  |  | 8743 | -0.281 | -0.0191 | No |
| 101 | NEAT1 |  |  | 8775 | -0.334 | 0.0024 | No |
Table: GSEA details [plain text format]

  

Fig 2: LARSSON\_STATE\_6: Random ES distribution      
 Gene set null distribution of ES for **LARSSON\_STATE\_6**
